# Antibiotic prophylaxis for surgical procedures: a scoping review

**DOI:** 10.26633/RPSP.2021.62

**Published:** 2021-05-26

**Authors:** Eva Brocard, Ludovic Reveiz, Jean-Philippe Régnaux, Veronica Abdala, Pilar Ramón-Pardo, Ana del Rio Bueno

**Affiliations:** 1 Ecole des Hautes Etudes en Santé Publique (EHESP) Rennes France Ecole des Hautes Etudes en Santé Publique (EHESP), Rennes, France; 2 Pan American Health Organization Washington, D.C. United States of America Pan American Health Organization, Washington, D.C., United States of America

**Keywords:** Antibiotic prophylaxis, surgical procedures, operative, surgical wound infection, Profilaxis antibiótica, procedimientos quirúrgicos operativos, infección de la herida quirúrgica, Antibioticoprofilaxia, procedimentos cirúrgicos operatórios, infecção da ferida cirúrgica

## Abstract

**Objectives.:**

To map the current evidence on surgical antibiotic prophylaxis (SAP) administration and identify knowledge gaps in the literature available in this field.

**Methods.:**

The PubMed, Cochrane Library, Epistemonikos, and Health Systems Evidence databases were searched from January 2015 to March 2020 for systematic reviews published in English, French, Portuguese, and Spanish.

**Results.:**

Eighty-three systematic reviews were included, the quality of the reviews was assessed using AMSTAR 2, and data were extracted for all primary outcomes. Perioperative antibiotic administration, the use of first generation cephalosporins, and surgical site infection (SSI) were the most commonly reported for timing of antibiotic administration, drug class, and primary outcome, respectively. Findings showed that, overall, SAP may reduce SSIs compared with a placebo or with no SAP. Results suggested that intraoperative SAP may lower SSI, while postoperative SAP did not show a statistically significant difference.

**Conclusions.:**

Findings have confirmed the role of SAP in reducing postoperative SSI across various surgeries and do not support the use of antibiotics after surgery to prevent infections. The findings of this scoping review have enhanced the evidence base that can inform decisions regarding the development of global guidelines for the prevention of SSI. However, high-quality systematic reviews and research reflecting diverse populations and settings are needed.

## RESUMEN

**Objetivos.:**

Trazar un mapa de la evidencia actual sobre la administración de profilaxis antibiótica quirúrgica e identificar lagunas de conocimiento en la bibliografía disponible en este campo.

**Métodos.:**

Se realizaron búsquedas en las bases de datos PubMed, Cochrane Library, Epistemonikos y Health Systems Evidence desde enero del 2015 hasta marzo del 2020 para obtener revisiones sistemáticas publicadas en inglés, francés, portugués y español.

**Resultados.:**

Se incluyeron ochenta y tres revisiones sistemáticas, se evaluó la calidad de las revisiones con AMSTAR 2 y se extrajeron los datos de todos los resultados primarios. Se notificó con mayor frecuencia la administración de antibióticos perioperatorios, el uso de cefalosporinas de primera generación y la infección de sitio quirúrgico en relación con los tiempos de administración de los antibióticos, el tipo de medicamento y el resultado principal, respectivamente. Los resultados demostraron que, en términos generales, la profilaxis antibiótica quirúrgica puede reducir la infección de sitio quirúrgico en comparación con un placebo o la falta de profilaxis. Los resultados sugirieron que la profilaxis antibiótica transoperatoria puede reducir la infección de sitio quirúrgico, si bien la profilaxis antibiótica posoperatoria no mostró una diferencia estadísticamente significativa.

**Conclusiones.:**

Los resultados confirman la función de la profilaxis antibiótica quirúrgica en la reducción de la infección posoperatoria de sitio quirúrgico en diversas operaciones quirúrgicas y no avalan el uso de antibióticos después de la cirugía para prevenir infecciones. Los resultados de esta revisión exploratoria han contribuido a la base empírica que puede fundamentar decisiones relacionadas con la formulación de directrices mundiales para la prevención de infección de sitio quirúrgico. Sin embargo, se necesitan revisiones sistemáticas e investigación de calidad que representen poblaciones y entornos diversos.

## RESUMO

**Objetivo.:**

Mapear as evidências atuais em administração de antibioticoprofilaxia cirúrgica e identificar as lacunas de conhecimento na literatura existente nesta área.

**Métodos.:**

Foram realizadas buscas nos repositórios PubMed, Cochrane Library, Epistemonikos e Health Systems Evidence de janeiro de 2015 a março de 2020, limitadas a revisões sistemáticas publicadas em espanhol, francês, inglês e português.

**Resultados.:**

Oitenta e três revisões sistemáticas foram incluídas. A qualidade das revisões foi avaliada com o uso do instrumento AMSTAR 2. Foram extraídos dados para todos os desfechos primários. O período perioperatório foi o momento de aplicação da antibioticoprofilaxia mais comumente relatado; cefalosporinas de primeira geração, a classe terapêutica mais comumente utilizada; e infecção do sítio cirúrgico (ISC), o desfecho primário mais comumente descrito. Os achados desta revisão demonstram que, em geral, a antibioticoprofilaxia cirúrgica pode reduzir a ocorrência de ISC quando comparada ao placebo ou à não realização de antibioticoprofilaxia. Os resultados sugerem que a antibioticoprofilaxia cirúrgica transoperatória pode reduzir a ocorrência de ISC, embora a profilaxia pós-operatória não tenha demonstrado diferença estatisticamente significativa.

**Conclusões.:**

Este estudo confirma o papel da antibioticoprofilaxia cirúrgica em reduzir ISC pós-operatória em diversos procedimentos cirúrgicos, mas não respalda o uso de antibióticos no pós-operatório para prevenir infecções. Os resultados desta revisão de escopo reforçam o corpo de evidências para subsidiar decisões ao se elaborar diretrizes globais para a prevenção de ISC. Porém, são necessárias revisões sistemáticas de alta qualidade e pesquisas em populações e cenários diversos.

Antimicrobial resistance (AMR) is a global health challenge that does not respect geopolitical borders. Although a natural phenomenon, AMR has accelerated in the past few decades due to several factors such as excessive prescriptions, inappropriate consumption of antibiotics (1), poor hygiene practices, and extensive use of antimicrobials in livestock production (2). AMR has detrimental consequences on the health of individuals in both developed and developing countries and undermines considerably the ability to prevent and treat infectious diseases such as tuberculosis, HIV, and malaria. Globally, a 2014 report estimates that the number of AMR-related deaths could be as high as 700 000 per year (3). According to a recent study (4) in 2015 in the European Union, an estimated 33 000 deaths were attributable to infections with antibiotic-resistant bacteria, while the United States Centers for Disease Control and Prevention (CDC) (5) reported that more than 2.8 million antibiotic-resistant infections occur annually and more than 35 000 people die as a result. The spread of AMR is not only a threat to human health but is also a burden on healthcare systems and countries’ economies. Due to weak AMR surveillance and lack of information about its spread, estimating the cost and economic impact of AMR is challenging. Therefore, based on estimates and AMR impacts, the World Bank released a report (6) that projected a 1.1% fall in the global output by 2050 in an optimistic case scenario, while in a more pessimistic scenario, the global output losses would reach 3.8% by 2050.

Misuse (excessive or inappropriate use) of antibiotics is the main driver of the increasing AMR (7). Antibiotics can be given to treat an infection and, as a precaution, to prevent an infection, known as antibiotic prophylaxis (AP). The three most common indications for AP in immunocompetent patients are infections and diseases unrelated to surgical procedures (i.e., recurrent cellulitis, meningococcal disease, or recurrent urinary tract infections (UTIs)), prior to invasive dental procedures (infective endocarditis) (8), and to prevent surgical site infections (SSI). SSIs are potential complications that can occur after any type of surgical procedure and are among the most preventable healthcare-acquired infections. They are defined by the CDC as superficial (involving skin) or deep (involving soft tissues, organs) infections occurring within 30 days post surgery (9).

SSIs cause a detrimental burden on the health of communities but also on health systems. To date, no formal guidelines on the use of AP for non-surgical procedures are available. In 2016, a Systematic Reviews Expert Group convened by the World Health Organization (WHO) produced guidelines for the prevention of SSIs (10). Four of their 29 recommendations provide instructions on AP to prevent SSIs: antibiotics must be administered between 60 and 120 minutes before the surgery; no postoperative prolongation after any type of surgery; as well as no extension in case of wound drain; and patients undergoing elective colorectal surgery should be given preoperative oral antibiotics combined with mechanical bowel preparation. Furthermore, “*Tratamiento de las enfermedades infecciosas 2020-2022*,” a publication by the Pan American Health Organization (PAHO), the WHO Regional Office for the Americas, provides comprehensive recommendations on the use of prophylactic antibiotics for adults and children undergoing surgical procedures (11). Despite the production of recent guidelines, surgical antibiotic prophylaxis (SAP) misuses persist and continue to increase the risk for acquisition of SSIs. To reduce this, it is necessary to study the most recent evidence available on SAP administration practices. This scoping review aims to report existing knowledge and to map the research conducted so far on SAP, as well as to identify any gaps in the research.

## MATERIALS AND METHODS

The protocol was developed using the scoping review methodological framework proposed by the Joanna Briggs Institute (12) and followed the guidelines of the Preferred Reporting Items for Scoping Reviews (PRISMA-ScR) to guide reporting.

### Eligibility criteria

Inclusion criteria were framed using PICO elements (13) (see Table 1). Studies were included if they were systematic reviews (SR), irrespective of the design of the studies included (randomized controlled trials, observational, cohorts), published between January 2015 and March 2020, and reported on the administration, prescription or use of AP for surgical procedures. For the present review, AP was defined as antibiotics that were provided preoperatively, or preoperatively and postoperatively, for preventing postoperative infectious complications. Reviews about antivirals were not included in the scoping review.

**TABLE 1.** PICOS inclusion criteria applied to potential eligible searched citations

Element	Criterion
Population	Both adults and children (<18 years), patients undergoing surgical intervention
Intervention	Single or multi-dose antibiotic given as prophylaxis either before, during, or after surgery
Comparator	Comparators investigated in systemic review, such as (but not limited to) placebo, no treatment, another antibiotic regimen, for example
Outcomes	Only primary outcomes reported by the systematic reviews were included
Study design	Systematic reviews

***Source:*** Prepared by the authors.

### Information sources

The PubMed, Cochrane Library, Epistemonikos, and Health Systems Evidence databases were searched for SRs published within the last five years. The references of the included studies were also searched.

### Search

The final search strategy was developed and adapted to each database (see Appendix 1 in supplementary material). In order to ensure the capture of all the relevant titles that met the inclusion criteria of the study, the scoping included a manual search using Google Scholar. No additional SRs were retrieved from the manual search. Studies published in English, French, Portuguese, and Spanish were considered.

### Selection of sources of evidence

Studies were selected by one author (EB) and a second author (LR) verified the selection. Any discrepancy was discussed by the team. The screening process followed two stages. First, we reviewed the titles and abstracts of the retrieved studies to identify potentially eligible articles according to the inclusion criteria. The full-text articles were reviewed independently by two authors for final inclusion. Discrepancies were resolved by discussion or by a third author if no consensus was reached.

### Data extraction process

A data extraction form was piloted using Excel. The form was tested by extracting data on a sample of five studies and modified on feedback from the team. The extraction of the data was conducted by one author (EB) and verified by a second author (LR). Differences in extraction between the two reviewers were resolved by discussion and consensus.

### Data items

We extracted the following key information: general characteristics of studies (first author’s name, year of publication, scientific title), number of studies included, sample sizes and details of participants (number, age group, gender), type of prophylaxis, drug, type of surgical procedure classified per PAHO categories (11), comparisons, primary outcomes measured, and key findings.

### Critical appraisal of the systematic reviews

The quality and risk of bias in included SRs were critically appraised using the AMSTAR 2 tool, which is a validated and reliable tool (14). Two reviewers independently assessed a sample of included SRs (10.8%) with the AMSTAR tool. A good agreement (79%) between the two raters was obtained. Assessments were performed by one author (EB). Uncertainties were resolved in discussions with a second author (JPR).

### Synthesis of results

Descriptive statistics were used to summarize the data and present the results. Categorical variables were presented as number and percentage, and continuous variables were presented as mean and range. Contributing characteristics such as numbers of SRs associated with the timing of prescribed antibiotics, main antibiotics reported, as well as the main results were presented in a synthesized table in the results section. Results for each SR are presented in detail based on the characterization of the included studies, and an evidence map was also developed with the support of the Latin American and Caribbean Center on Health Sciences Information at PAHO.

## RESULTS

### Selection of systematic reviews

**FIGURE 1.**
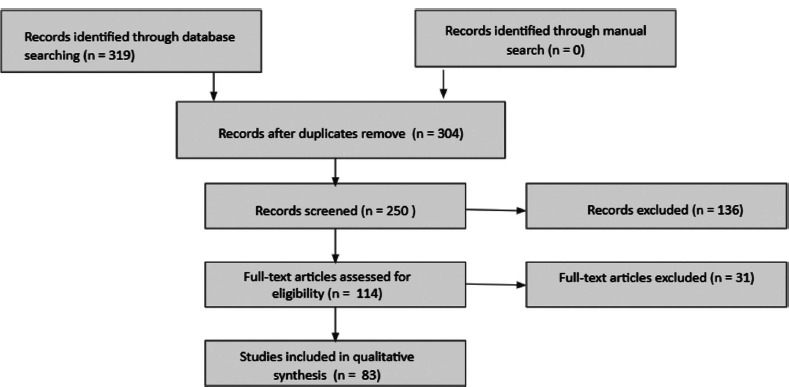
PRISMA flowchart of study selection

***Source:*** Prepared by the authors from the study results.

The preliminary search yielded 319 studies that were SRs published in the last five years; 15 duplicates were removed. After excluding 54 irrelevant titles, 250 abstracts were reviewed. Of those, full text was screened for 114 records and 83 met the inclusion criteria (Figure 1). Reasons for exclusion were: (i) use of antibiotic prophylaxis for non-surgical procedures, (ii) antimicrobial prophylaxis with antivirals, and (iii) duplicates.

### Characteristics of included systematic reviews

Among the SRs included, 8 (9.6%) were published in 2015, 13 (15.6%) in 2016, 11 (13.2%) in 2017, 17 (20.4%) in 2018, 29 (34.9%) in 2019, and 5 (6.0%) for 2020 so far. Of the SRs, 61 (73.5%) performed a meta-analysis, while the remaining 23 (26.5%) did not perform a quantitative analysis, mostly due to the small number of studies included in their review. Information about the sample size of studies included in the SR was obtained for all (100%) SRs. The lowest number of studies included in an SR was 0 (no study met inclusion criteria for an SR) and the highest number was 74. For the sample sizes, the minimum was 0 participants included (no study met inclusion criteria for an SR) to a maximum of 900 000 participants. In terms of participants, except for conditions and surgeries exclusively related to one gender, such as breast surgery and cesarean section for women and prostate biopsies for men, all the other SRs included both female and male patients. Only one study (15) presented data on SAP for pediatric patients undergoing cardiac surgery; all other studies reported on adult population.

### Quality assessment of the systematic reviews

After evaluation, 27 (32.5%) SRs were rated with a high quality score, 27 (32.5%) with moderate quality score, 14 (16.9%) with low quality score, and 15 (18.1%) were rated with a critically low quality score (see Appendix 2 in supplementary material). If more than half the reviews (65%) had a quality score of high or moderate, most of the SRs presented great levels of heterogeneity in the studies they included. Twenty-three SRs (27.7%) did not conduct a meta-analysis due to heterogeneity levels but also the small number of studies. Indeed, 33 (39.7%) reviews included fewer than 10 studies. Furthermore, only a few of them were able to report evidence only from randomized controlled trials, whereas most of the SRs included non-randomized trials, downgrading the quality of the evidence. The evidence map developed gives an overview of the quality of the 83 SRs included in the study, as bubbles. The map is available from the following link: https://public.tableau.com/profile/caio.fabio.schlechta.portella#!/vizhome/Antibioticos/Planilha1?publish=yes

**TABLE 2.** Summary of main findings from the systematic reviews, by the timing of surgical antibiotic prophylaxis administration and the main antibiotics reported

Timing of SAP	No. of SRs	Main antibiotics reported	Key overall results
Preoperative	16	1st and 2nd generation cephalosporin, β-lactams, aminoglycosides, and 2nd generation fluoroquinolones	Compared with placebo or no treatment, preoperative SAP was found to lower SSIs for various surgeries (neurosurgery, cesarean section, urological). One SR investigated appropriate timing of administration and 120 min or less was found to lower postoperative SSI, while more than 120 min increased them.
Intraoperative	10	1st generation cephalosporin, vancomycin, and gentamicin	Results suggest that intraoperative SAP lowers SSI rates and wound infections compared with no antibiotics or a placebo.
Postoperative	10	Amoxicillin/clavulanic, 1st and 2nd generation cephalosporin	Mixed results were found about the impact of postoperative SAP compared with no antibiotics or placebo on SSI, wound infections, and other outcomes. Only 1 SR found a statistically significant reduction in SSI with post SAP, while 6 SRs found none. Results of 3 SRs suggest that post SAP may probably reduce fistula rates, endophthalmitis, and anaphylaxis.
Perioperative	44	1st and 2nd generation cephalosporin, vancomycin, gentamicin, fluoroquinolones, penicillin	High heterogeneity in terms of results. Results corroborated preoperative findings showing that SAP lowers SSI compared with placebo or no treatment. However, findings suggest that SAP prolongation/postoperative did not show a statistically significant difference in terms of SSI and wound infections, compared with preoperative SAP alone.

***Note:*** SAP, surgical antibiotic prophylaxis; SR, systematic review; SSI, surgical site infection.

***Source:*** Prepared by the authors from the study results.

### Results of individual systematic reviews

The search showed at least one SR published within the last five years on the use of AP for all major surgical categories as stated in the PAHO guidelines “*Tratamiento de las enfermedades infecciosas 2020-2022*,” except for the categories of thoracic surgery, surgical abortion, and esophageal and obesity surgery. The highest number of SRs was retrieved for the category on orthopedic surgeries (20 SRs (24.1%)).

Various antibiotic regimens were reported among the 83 SRs. They have been studied alone, in comparison with no SAP administration or a placebo, or against another antibiotic type, and as part of a combination with another antibiotic or intervention. The most-reported antibiotics in the literature were first and second generation cephalosporins (cefazolin and cefuroxime). The most-listed primary outcome was SSIs (51.8%). Furthermore, five primary outcomes (SSIs, wound infections, adverse events, length of hospital stay, and UTIs) accounted for three-quarters (74.6%) of the reported primary outcomes.

Among the four categories of administration of antibiotic timing (preoperative, intraoperative, postoperative, perioperative), the majority (44 (53.0%)) of the SRs included studies that reported the administration of antibiotics perioperatively (including at least two different timings of administration). Furthermore, 16 (19.3%) SRs reported information on preoperative antibiotic prophylaxis, while only 10 (12.0%) studied intraoperative antibiotic administration, and 10 (12.0%) others focused only on prolongation of antibiotic prophylaxis post surgery. Finally, 2 (2.4%) SRs did not provide any detail about the timing of administration of antibiotics, while for 1 (1.2%) SR it was not relevant, as no studies met the inclusion criteria of the review.

The results of most of the included SRs showed a statistically significant reduction of SSI for patients that received preoperative SAP for various surgeries, compared with the absence of antibiotics and/or placebo. Results on intraoperative SAP suggested that their administration may have lowered SSI as well. However, prolonged and postoperative antibiotic prescription did not show statistically significant reduction of SSI or other outcomes such as UTIs, and in some cases even increased UTIs. As mentioned before, most of the SRs (*n* = 44, 53.0%) did not look at one specific timing of administration but included studies in which antibiotics were given either pre-, intra-, or post-surgery or at a combination of different moments. Therefore, conclusions of those heterogeneous results are that a single dose of SAP showed a statistically significant reduction of several primary outcomes (mostly SSI and wound infections), compared with no antibiotics or a placebo. Due to the extensive evidence retrieved, a summary of the main results is presented in Table 2. The findings of each SR included in the scoping review are presented in Appendix 3 in the supplementary material.

## DISCUSSION

This scoping review has provided a starting point in an area of increasing interest internationally, by scoping the literature to identify existing clinical practices related to SAP. This scoping review shows that SAP has been well documented for certain categories of surgical procedures and more specifically orthopedic surgeries, which accounted for 20 of the 83 studies included in the review. Findings showed that antibiotics were administered at different times in relation to the surgery and very few studies reported the dosage of the antibiotics prescribed.

### Generalization and applicability of results

This present scoping review shows that overall, preoperative SAP lowers postoperative SSIs and should be preferred to the absence of AP. This review brought to light inconclusive evidence on the effect of prolonged and/or postoperative AP for the prevention and reduction of SSIs. However, one SR included in the scoping review (16) and one survey study conducted in Italy (17) found critical compliance problems of clinical practices with SAP guidelines. The main reasons stated for the lack of adherence to guidelines were the absence of clarity in SAP indications. The heterogeneity of antibiotic regimens reported in the scoping review shows that the appropriate use of SAP remains a significant challenge. As new guidelines have been produced by WHO in 2016 and by PAHO in 2019, we identify the need to study compliance in clinical practice, as well as the reasons behind inappropriate administration by surgeons, in order to improve and optimize the delivery of SAP.

To our knowledge, no other scoping review has been conducted and published before on SAP for various surgical procedures. With this in mind, this scoping review is an evidence-informed overview of antibiotic regimens given as prophylaxis for surgical procedures. The results of the review are in line with the guidelines developed by PAHO and WHO, recommending preoperative AP to prevent SSIs. However, caution must be applied in the generalization of the results, as many gaps for external validity have been identified with this review. Although this scoping review provides considerable evidence supporting the use of SAP, it lacks clear evidence related to the optimal antibiotic regimen (class, dose, route, and timing) for each surgical procedure as well as for targeted population (children vs. adults). The results show the great heterogeneity of SAP prescription and use and provide similar findings to a literature review of 50 SRs and meta-analysis (18), which compared SAP administration practices to Australian national guidelines on antibiotic prophylaxis. The review showed that single-dose first-generation cephalosporin seemed to be the antibiotic regimen of choice for various types of surgery. However, some limitations were found in terms of providing the specific optimal antibiotic regimen per surgery. Another challenge for the generalization of the results includes the variety of settings (countries, type of care facility) reported. Furthermore, the findings are for the most part inapplicable to pediatric patients due to the lack of evidence retrieved from this present scoping review. In addition, the majority of the reviews either did not provide information about the routes of administration of the antibiotics or reported it but did not study the optimal route of antibiotics for each surgery or population. Finally, head-to-head comparisons of antibiotics should also be further investigated, as very few SRs in the scoping review directly compared two classes of antibiotics but instead had for comparator a placebo or the absence of treatment.

Despite the extensive evidence retrieved, this scoping review has shown the existence of research gaps related to the use of SAP for certain surgical procedures, populations, such as pediatric patients, and operative settings, which should be studied alone, as recommendations on SAP administration will differ. Furthermore, most of the SRs presented high levels of heterogeneity in the studies they included. These levels were due to the variations in the settings where the studies were conducted, the antibiotic regimen (dosage, type of antibiotic, timing), and the comparators. However, for certain procedures that lacked evidence, such as liver-kidney transplant (2 SRs), evidence was found through the search. Indeed, the search yielded five studies (19–23) that looked into antiviral prophylaxis. Those SRs studied the effectiveness of ganciclovir and/or valganciclovir in the prevention of cytomegalovirus disease for people undergoing solid organ transplant. None of the five SRs showed a real significant difference in cytomegalovirus infection and disease between patients who were given valganciclovir (regardless of high or low dose, pre/intra/post-use) compared with those who were not. For the other types of surgery, the search did not yield results related to prescriptions of other antimicrobials.

### Limitations and strengths

The scoping review limited the search to only include publications within the last five years. However, we anticipate that the search has captured most relevant reviews and thus provides a good overview of currently available evidence on the use of AP for surgical procedures. AMSTAR 2 presented methodological limitations due to the unequal importance of the reporting items. Future reviews may consider the other assessment tool—risk of bias in systematic reviews (ROBIS)—in addition to these quality assessments.

The strength of this review is its comprehensive scope, which included a wide definition of AP, as well as the inclusion of studies published in four languages, which enabled capture of instrumental information on the use, regimen, dosage, and timing of the administration of antibiotics for people undergoing surgery. We were able to rapidly gather information on SAP under almost all the surgical categories stated in the PAHO guidelines (11).

### Conclusion

The results of this scoping review have enhanced the evidence base that can inform decisions regarding recommendations for the administration of AP for surgical procedures. Findings have confirmed the role of SAP in reducing postoperative SSI across various surgeries. Results do not support the use of antibiotics after surgery to prevent infections. This scoping review has identified gaps in the current research evidence on SAP that need to be addressed in order to strengthen evidence and provide adequate clinical recommendations. Therefore, high-quality SRs and research reflecting diverse populations and settings are needed.

## Disclaimer.

Authors hold sole responsibility for the views expressed in the manuscript, which may not necessarily reflect the opinion or policy of the *RPSP/PAJPH* and/or PAHO.

## References

[1] 1. Cecchini M, Langer J, Slawomirski AL. Antimicrobial Resistance in G7 Countries and Beyond: Economic Issues, Policies and Options for Action. Paris: OECD; 2015. Available from: https://www.oecd.org/els/health-systems/Antimicrobial-Resistance-in-G7-Countries-and-Beyond.pdf Accessed 2020 Aug 5.

[2] 2. Van Boeckel TP, Brower C, Gilbert M, Grenfell BT, Levin SA, Robinson TP et al. Global trends in antimicrobial use in food animals. Proc Natl Acad Sci U S A. 2015;112(18):5649–54. 10.1073/pnas.1503141112 PMC4426470 25792457

[3] 3. The Review on Antimicrobial Resistance, chaired by Jim O’Neill. Antimicrobial Resistance: Tackling a crisis for the health and wealth of nations. Dec 2014. Available from: http://www.jpiamr.eu/wp-content/uploads/2014/12/AMR-Review-Paper-Tackling-a-crisis-for-the-health-and-wealth-of-nations_1-2.pdf Accessed 2020 Aug 5.

[4] 4. Cassini A, Högberg LD, Plachouras D, Quattrocchi, A, Hoxha A, Simonsen GS et al. Attributable deaths and disability-adjusted life-years caused by infections with antibiotic-resistant bacteria in the EU and the European Economic Area in 2015: a population-level modeling analysis. Lancet Infect Dis. 2019;19(1):56–66. 10.1016/S1473-3099(18)30605-4 PMC6300481 30409683

[5] 5. United States Centers for Disease Control and Prevention. Antibiotic Resistance Threats in the United States. Atlanta: CDC; 2019. Available from: https://www.cdc.gov/drugresistance/pdf/threats-report/2019-ar-threats-report-508.pdf Accessed 2020 Aug 5.

[6] 6. Jonas OB, Irwin A, Berthe FCJ, Le Gall FG, Marquez PV. Drug-resistant infections : a threat to our economic future, final report. HNP/Agriculture Global Antimicrobial Resistance Initiative. Washington DC: World Bank Group; 2017. Available from: http://documents.worldbank.org/curated/en/323311493396993758/final-report Accessed 2020 Aug 5.

[7] 7. Enzler MJ, Berbarii E, Osmon DR. Antimicrobial prophylaxis in adults. Mayo Clinic Proceedings. 2011;86(7):686–701. 10.4065/mcp.2011.0012 PMC3127564 21719623

[8] 8. Thornhill MH, Gibson TB, Cutler E, Dayer MJ, Chu VH, Lockhart PB, et al. Antibiotic Prophylaxis and Incidence of Endocarditis Before and After the 2007 AHA Recommendations. J Am Coll Cardiol. 2018;72(20):2443–54. doi: 10.1016/j.jacc.2018.08.2178 10.1016/j.jacc.2018.08.2178 30409564

[9] 9. Mangram AJ, Horan TC, Pearson ML, Silver LC, Jarvis WR. Guideline for Prevention of Surgical Site Infection, 1999. Centers for Disease Control and Prevention (CDC) Hospital Infection Control Practices Advisory Committee. Am J Infect Control. 1999;27(2):97–132. 10196487

[10] 10. World Health Organization. Global Guidelines for the Prevention of Surgical Site Infection. Geneva: WHO; 2016. Available from: https://apps.who.int/iris/bitstream/handle/10665/250680/9789241549882-eng.pdf Accessed 2020 Aug 5.

[11] 11. Organización Panamericana de la Salud. Tratamiento de las enfermedades infecciosas 2020-2022. Octava edición. Washington DC: OPS; 2019. Available from: https://iris.paho.org/handle/10665.2/51695 Accessed 2020 Aug 5.

[12] 12. Peters MDJ, Godfrey C, McInerney P, Munn Z, Tricco AC, Khalil, H. Chapter 11: Scoping Reviews (2020 version). In: Aromataris E, Munn Z (Editors). JBI Manual for Evidence Synthesis. [Adelaide]: JBI; 2020. Available from: https://synthesismanual.jbi.global

[13] 13. McKenzie JE, Brennan SE, Ryan RE, Thomson HJ, Johnston RV, Thomas J. Chapter 3: Defining the criteria for including studies and how they will be grouped for the synthesis. In: Higgins JPT, Thomas J, Chandler J, Cumpston M, Li T, Page MJ, et al. Cochrane Handbook for Systematic Reviews of Interventions. [London]:Cochrane; 2020. Version 6.1. Available from: https://training.cochrane.org/handbook/current/chapter-03 Accessed 2020 Aug 5.

[14] 14. Shea BJ, Reeves BC, Wells G, Thuku M, Hamel C, Moran J, et al. AMSTAR 2: a critical appraisal tool for systematic reviews that include randomised or non-randomised studies of healthcare interventions, or both. BMJ. 2017;358:j4008. doi: 10.1136/bmj.j4008 10.1136/bmj.j4008 PMC5833365 28935701

[15] 15. Jaworski R, Kansy A, Dzierzanowska-Fangrat K, Maruszewski B. Surgery: Where Are We and Where Do We Go? A Systematic Review. Surg Infect (Larchmt). 2019;20(4):253–60. 10.1089/sur.2018.272 30762492

[16] 16. Ghobrial GM, Cadotte DW, Williams K Jr, Fehlings MG, Harrop JS. Complications from the use of intrawound vancomycin in lumbar spinal surgery: a systematic review. Neurosurg Focus. 2015;39(4):E11. doi: 10.3171/2015.7.FOCUS15258 10.3171/2015.7.FOCUS15258 26424335

[17] 17. Testa M, Stillo M, Giacomelli S, Scoffone S, Argentero PA, Farina EC, et al. Appropriate use of antimicrobial prophylaxis: an observational study in 21 surgical wards. BMC Surg. 2015;15:63. doi: 10.1186/s12893-015-0048-7 10.1186/s12893-015-0048-7 PMC4434534 25968324

[18] 18. Lerano C, Thursky K, Marshall C, Koning S, James R, Johnson S, et al. Appropriateness of Surgical Antimicrobial Prophylaxis Practices in Australia. JAMA Netw Open. 2019;2(11):e1915003. doi: 10.1001/jamanetworkopen.2019.15003 10.1001/jamanetworkopen.2019.15003 PMC6902799 31702804

[19] 19. AlDabbagh MA, Gitman MR, Kumar D, Humar A, Rotstein C, Husain S. The role of antiviral prophylaxis for the prevention of Epstein-Barr virus-associated posttransplant lymphoproliferative disease in solid organ transplant recipients: a systematic review. Am J Transplant. 2017;17:770–81. doi: 10.1111/ajt.14020 10.1111/ajt.14020 27545492

[20] 20. Xin W, Hui Y, Xiaodong Z, Xiangli C, Shihui W, Lihong L Effectiveness of Valganciclovir 900mg Versus 450mg for Cytomegalovirus Prophylaxis in Renal Transplantation: A Systematic Review and Meta-Analysis. J Pharm Pharm Sci. 2017;20:168–83. doi: 10.18433/J3805B 10.18433/J3805B 28719361

[21] 21. Hui Y, Xiangli C, Xin W, Shuang Q, Lihong L. Clinical Outcomes with Antiviral Prophylaxis or Preemptive Therapy for Cytomegalovirus Disease after Liver Transplantation: A Systematic Review and Meta-Analysis. J Pharm Pharm Sci. 2017;20:15–27. doi: 10.18433/J3RC90 10.18433/J3RC90 28459662

[22] 22. Wong DD, van Zuylen WJ, Craig ME, Rawlinson WD. Systematic review of ganciclovir pharmacodynamics during the prevention of cytomegalovirus infection in adult solid organ transplant recipients. Rev Med Virol. 2019;29(2):e2023. doi: 10.1002/rmv.2023 10.1002/rmv.2023 30556615

[23] 23. Hwang SD, Lee JH, Lee SW, Kim JK, Kim MJ, Song JH. Effect of Low-Dose Vs Standard-Dose Valganciclovir in the Prevention of Cytomegalovirus Disease in Kidney Transplantation Recipients: A Systemic Review and Meta-Analysis. Transplant Proc. 2018;50(8):2473–8. doi: 10.1016/j.transproceed.2018.01.023 10.1016/j.transproceed.2018.01.023 29871773

